# Intravitreal Aflibercept for Patients with Acute Nonarteritic Anterior Ischemic Optic Neuropathy: A Retrospective Trial

**DOI:** 10.3390/jcm12144868

**Published:** 2023-07-24

**Authors:** Kai-Chun Cheng, Chien-Chih Chiu, Kuo-Jen Chen, Yo-Chen Chang

**Affiliations:** 1 Department of Ophthalmology, Kaohsiung Municipal Siaogang Hospital, Kaohsiung 812, Taiwan; pington64@gmail.com (K.-C.C.); 0870649@kmhk.org.tw (K.-J.C.); 2 Department of Ophthalmology, Kaohsiung Medical University Hospital, Kaohsiung 807, Taiwan; 3 Department of Ophthalmology, School of Medicine, College of Medicine, Kaohsiung Medical University, Kaohsiung 807, Taiwan; 4 Department of Biotechnology, Kaohsiung Medical University, Kaohsiung 807, Taiwan; cchiu@kmu.edu.tw; 5 Center for Cancer Research, Kaohsiung Medical University, Kaohsiung 807, Taiwan; 6 Department of Medical Research, Kaohsiung Medical University Hospital, Kaohsiung 807, Taiwan; 7 Department of Biological Sciences, National Sun Yat-sen University, Kaohsiung 804, Taiwan; 8 Graduate Institute of Medicine, College of Medicine, Kaohsiung Medical University, Kaohsiung 807, Taiwan

**Keywords:** nonarteritic anterior ischemic optic neuropathy (NAION), intravitreal aflibercept, visual field, retinal nerve fiber layer thickness (RNFLT), optical coherence tomography

## Abstract

Purpose: This study aimed to investigate whether intravitreal aflibercept was safe and effective in patients with acute nonarteritic anterior ischemic optic neuropathy (NAION). Methods: This was a chart study of 25 individuals with acute NAION (25 eyes). An intravitreal injection of 2 mg/0.05 mL of aflibercept was administered to fifteen participants. The remaining ten patients in the control group were given standard care. The researchers measured the initial visual acuity, retinal nerve fiber layer thickness (RNFLT), and automated perimetry. During the follow-up period, the researchers measured the final visual acuity, RNFLT, automated perimetry, and side effects. Results: Visual acuity and visual field assessment were significantly improved in the study group, and optical coherence tomography testing demonstrated significant disc edema resolution. The therapy results differed significantly between the two groups regarding visual outcomes (F = 0.027, *p =* 0.039) and RNFLT decrease (F = 5.507, *p* = 0.003). However, the difference in visual field alterations was not significant (F = 0.724, *p =* 0.387). Conclusions: Intravitreal injection of aflibercept can significantly improve visual acuity and resolve disc edema in patients with acute NAION. Intravitreal aflibercept may be an alternative treatment for acute NAION. However, a large series investigation is needed to assess the long-term therapeutic benefit and safety of intravitreal aflibercept in patients with acute NAION.

## 1. Introduction

Nonarteritic anterior ischemic optic neuropathy (NAION) is believed to arise from an ischemic process within the retrolaminar part of the optic nerve head, which is predominantly furnished by the short posterior ciliary arteries [1]. This condition causes the sudden onset of painless visual loss in adults older than 50 [2]. There is no gender predisposition in NAION [3,4,5,6,7]. However, certain studies have indicated that the incidence of NAION tends to be slightly higher in males than in females [8,9,10]. According to a literature review, there is no specific preference for the occurrence of NAION in either eye. NAION can affect either the left or the right eye, and in most cases, it only affects one eye. However, it is possible for both eyes to be affected in the same patient [11]. In the Ischemic Optic Neuropathy Decompression Trial (IONDT), it was observed that approximately 15% of patients experienced the development of NAION in their other eye within 5 years [12]. There is no established therapy for NAION-associated decreased vision [13]. However, numerous medical and surgical therapies have been suggested for the treatment of NAION. Given the uncertain understanding of the underlying pathophysiology of NAION, many of these treatment options are empirical and encompass a broad range of agents that are hypothesized to target thrombosis, blood vessels, and optic disc edema or potentially possess neuroprotective properties. Several treatment options have been proposed for NAION, but their effectiveness remains uncertain. These options include optic nerve sheath decompression; subtenon injections of vasodilators; intravitreal injections of bevacizumab or triamcinolone; and administration of intravenous or topical intraocular pressure (IOP)-lowering agents, vasopressors, anticoagulants, oral corticosteroids, levodopa, carbidopa, and various others. Despite a broad range of agents and procedures that have been adopted to treat NAION, most approaches have not produced promising results, and some have demonstrated significant risk [14,15,16,17,18,19].

Topical application of 0.2% brimonidine tartrate has been suggested to possess potential neuroprotective properties. A study investigating its efficacy found no statistically significant difference in visual acuity when comparing patients who received the placebo versus those who received the drug. However, there were some nonsignificant trends indicating improved results in visual field tests among the treatment group [18].

Another treatment option that has been explored is the daily administration of 75–325 mg of aspirin. However, although aspirin is a good anticoagulant, studies have demonstrated that it does not provide any significant benefit in improving the visual outcomes of patients with NAION [15].

The application of steroids to treat NAION has been used clinically and is recommended by neurologists. However, the use of steroids for acute NAION is still controversial. An extensive, noncontrolled, retrospective study by Hayreh et al. demonstrated that oral steroid treatment was beneficial in patients with NAION who presented visual acuity less than 20/70 and were seen within two weeks of onset [20]. However, this retrospective study was not randomized and only included patients with a baseline visual acuity of 20/70 or worse, making it challenging to determine whether oral steroids are an effective treatment for NAION. One retrospective study was carried out to evaluate the efficacy of intravenous steroids to treat NAION [21]. Intriguingly, the outcomes revealed that steroid application does not improve visual outcomes and potentially has harmful effects. Jonas demonstrated that intravitreal steroid injection yields a subtle improvement in visual acuity but may result in ocular complications, such as glaucoma [22]. One meta-analysis demonstrated that steroids do not significantly increase visual results in patients with NAION [23]. However, another study suggested that triamcinolone acetonide injections may have a potential role in reducing the duration of optic disc edema associated with NAION, which could potentially lead to a recovery of visual acuity. However, these injections have not shown a significant improvement in the visual field associated with NAION [24].

In addition to pharmacological interventions, researchers have explored various other treatment modalities for NAION. One such example is the study conducted by Arnold et al. [25], who investigated the use of hyperbaric oxygen therapy in 20 patients with acute NAION. The treatment involved administering hyperbaric oxygen at 2.0 atmospheres twice a day for a duration of 10 days. The visual outcomes of these patients were compared with a control group of 27 individuals who did not receive the treatment. However, the study by Arnold et al. did not demonstrate any beneficial effects of hyperbaric oxygen therapy in improving visual outcomes in NAION patients.

The IONDT aimed to evaluate the effectiveness of optic nerve decompression surgery as a treatment for acute NAION [14]. The underlying theory behind this surgical approach was that the optic nerve damage observed in NAION, which leads to progressive visual loss, is partially caused by intraneural swelling following ischemic events. This swelling could subsequently impair local vascular flow and axoplasmic transport within the optic nerve head. The hypothesis was that reducing the subarachnoid cerebrospinal fluid pressure around the optic nerve could potentially improve vascular function, axonal transport, or both, thereby reducing tissue injury in axons that is potentially reversible. Recruitment for the IONDT was terminated after two years, during which 119 patients underwent optic nerve decompression surgery, while 125 patients remained untreated. The decision to cease recruitment was made when the analysis of the gathered data did not demonstrate any significant benefits and may have even posed harm to the patients.

Transvitreal optic neurotomy has emerged as a potential therapeutic option for NAION. This procedure involves performing a pars plana vitrectomy, inducing posterior vitreous detachment, and making a stab incision at the nasal margin of the optic disc. The goal is to open the scleral canal and alleviate compression on the edematous optic nerve head. The rationale behind this approach is that if a compartment syndrome contributes to the pathophysiology of NAION, such a procedure could theoretically break the cycle of edema and vascular compression. Soheilian et al. [17] conducted a study to evaluate the outcomes of transvitreal neurotomy in patients with acute NAION and severe visual loss (ranging from counting fingers to 20/800) with an onset of symptoms prior to the surgery ranging from 15 to 90 days. The study reported visual acuity improvement in six patients, with a final range of counting fingers to 20/60. Importantly, this study had limitations, including a small sample size, potential sample bias (as it included patients with severe visual loss, making accurate pre- and postoperative visual measurements challenging), and a delayed onset of therapy. The authors emphasized the experimental nature of this procedure and recommended conducting a randomized clinical trial before considering it as a standard approach.

Aflibercept is a fully human, recombinant synthesis protein that binds as a ligand to vascular-endothelial-growth-factor (VEGF) family members, including VEGF-A and VEGF-B, and is indicated for the treatment of retinal edema due to several disease entities, such as retinal vein occlusion [26,27] and diabetic macular edema [28]. One case report recently applied this drug in patients with acute NAION and revealed significant amelioration in visual acuity and visual field [29]. However, this study included only one patient and was uncontrolled. Therefore, it is not easy to draw conclusions about this regimen. Thus, we performed retrospective, comparative, nonrandomized clinical interventional research to investigate changes in visual acuity (VA), visual field (VF), and retinal nerve fiber layer thickness (RNFLT) in patients with acute NAION managed with intravitreal aflibercept.

## 2. Methods

### 2.1. Data Collection

A retrospective chart study of 25 eyes from 25 individuals with acute NAION was performed. From January 2018 to March 2021, medical records for all patients with acute NAION treated at the Kaohsiung Municipal Siaogang Hospital (Kaohsiung, Taiwan) were evaluated. A total of 25 patients with new-onset NAION (25 eyes) were included in the retrospective comparative nonrandomized clinical interventional investigation. All the patients had clinical evidence of acute NAION that met the criteria for the Ischemic Optic Neuropathy Decompression Trial (IONDT) [14], which included a relative afferent pupillary defect, sudden vision loss within the previous 14 days, optic disc edema, and an abnormal VF consistent with optic neuropathy. The following were the inclusion criteria: (1) a diagnosis of NAION based on the IONDT, (2) new-onset NAION (less than 14 days), and (3) a normal macula on ocular examination.

The exclusion criteria were as follows: (1) any other etiology of optic nerve disease, (2) previous attack of NAION in the same eye, (3) history of glaucoma or clinical suspicion of glaucoma on presentation, (4) history of previous retinal disease that could affect visual acuity, and (5) patients with a follow-up time of less than six months. The study group consisted of 15 patients (nine men and six women; 15 eyes; eight right eyes), while the control group consisted of 10 patients (five men and five women; ten eyes; six right eyes).

**Figure 1:**
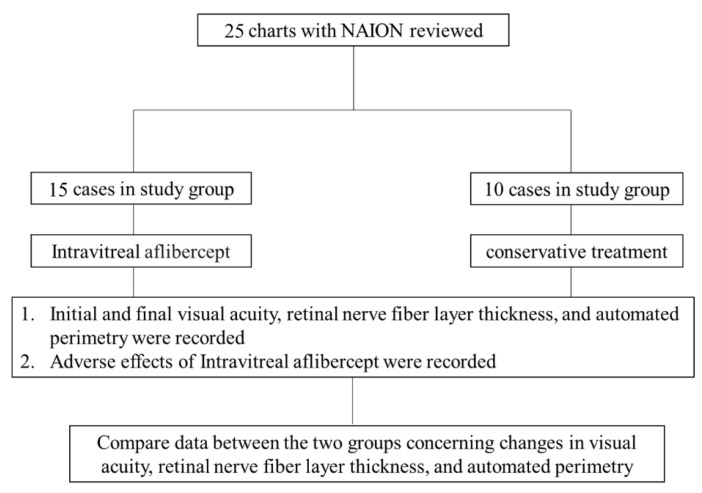
Flowchart of this study. This was a retrospective chart review of 25 consecutive patients with acute NAION. The study group was treated by intravitreal aflibercept injection. The control group received conservative treatment. Initial visual acuity, RNFL thickness, and automated perimetry were recorded. Final visual acuity, RNFL thickness, automated perimetry, and adverse events were recorded during the follow-up period.

Patients had no cataract surgery performed before, during, or after the intravitreal injection. Patients who agreed to participate in the trial agreed to receive a 2 mg intravitreal injection of aflibercept. The control group included patients who declined intravitreal aflibercept injection instead of conservative therapy. The flowchart for this investigation is shown in Figure 1. The research group’s average age was 61.20 ± 12.36 years, whereas the control group’s average age was 60.40 ± 8.66 years. The study group had a mean follow-up time of 229.73 ± 43.44 days, while the control group had a mean follow-up time of 258.90 ± 62.42 days (Table 1).

For statistical analysis, the best-corrected visual acuity was calculated from a Snellen chart and converted to the logarithm of the minimum angle of resolution (logMAR). All enrolled patients underwent a series of ophthalmic examinations of visual acuity at baseline and at repeated intervals afterward. A slit-lamp examination using an anterior segment, pneumotonometry (Full Auto Tonometer TX-F; Canon, NY, USA), dilated fundus examination using a Goldmann 3-mirror contact lens, optical coherence tomography (3D OCT-1 Maestro, Topcon, Tokyo, Japan) or indirect ophthalmoscopy of the optic nerves, and visual field testing with Humphrey automated static perimetry (HFA; Carl Zeiss Meditech, Inc., Dublin, CA, USA) were performed. Before receiving therapy, all patients provided informed consent, and this study was authorized by the Institutional Review Board of Kaohsiung Medical University Hospital in Kaohsiung, Taiwan (Approval No. KMUHIRB-E(I)-20200230). All protocols used in this study were also in adherence to the tenets of the Declaration of Helsinki.

The foregoing evaluations were performed on patients in the study group during the first week after injection, two times at two-weekly intervals and subsequently at monthly intervals. The same examinations were performed on the control group once a month. Once the disc edema had resolved and the visual acuity had stabilized or improved, the interval between follow-up examinations was lengthened. The key outcome measures were the Humphrey visual-field mean-deviation score, best-corrected visual acuity, RNFLT assessed using optical coherence tomography (OCT), and surgical complications.

### 2.2. Surgical Procedure

A sharp 30-gauge needle was used to inject 2 mg (0.05 mL) aflibercept (EYLEA; aflibercept solution for injection, Regeneron Pharmaceuticals, Inc., Tarrytown, NY, USA and Bayer HealthCare Pharmaceuticals, Berlin, Germany) into the vitreous cavity at 4 mm from the limbus for phakic patients and 3.75 mm for pseudophakic patients through the pars plana. The needle was withdrawn, and a sterile cotton-tipped applicator was used to pressure the injection site for a few seconds. Then, Tobradex ophthalmic suspension (tobramycin 0.3 percent, dexamethasone 0.1 percent; Alcon Laboratories Inc., Fort Worth, TX, USA) was administered. Patients were told to use Tobradex eye drops four times a day for three days after the injection.

### 2.3. Statistical Analysis

For continuous variables, independent-samples *t*-tests were used to compare baseline demographic and clinical data between the two groups, and chi-square tests were used to compare those data for categorical variables. For statistical analysis, visual acuity was transformed to the minimum angle of resolution (LogMAR). The mean SD was used to summarize the visual acuity, RNFLT, and mean deviation score of the Humphrey visual field during the baseline and follow-up visits. During follow-up, each patient’s change in visual acuity, RNFLT, and Humphrey visual-field mean-deviation score was computed. The study and control groups compared the mean changes across all patients and compared the changes during follow-up, utilizing between-group comparisons at the last visit with independent-samples *t*-tests.

Additionally, within each group, paired *t*-tests were used to compare baseline and end follow-up data (for visual acuity, RNFLT, and the Humphrey visual field mean deviation score). IBM SPSS Statistics for Windows, Version 20, was used to perform the statistical analyses (IBM SPSS Statistics for Windows, IBM Corporation, Armonk, NY, USA). Independent-samples and paired *t*-tests were used to analyze the data. A *p*-value of less than 0.05 was considered statistically significant.

## 3. Results

### 3.1. Baseline Characteristics

A total of 15 patients managed with intravitreal aflibercept and ten patients treated with conservative management between January 2018 and March 2021 qualified for analysis. The mean age was 61.20 ± 12.36 years in the study group, and the mean age was 60.40 ± 8.66 years in the control group. The average follow-up time was 229.73 ± 43.44 days for the study group and 258.90 ± 62.42 for the control group. All patients were followed up for at least six months. All patients in the study group underwent one injection of aflibercept between the initial and final follow-ups. The baseline characteristics of the groups were matched and are shown in Table 1. With respect to patient age, sex, mean follow-up period, baseline visual acuity, baseline RNFLT, or mean deviation, there were no statistically significant differences between the two groups.

### 3.2. Outcome Measures

**Figure 2:**
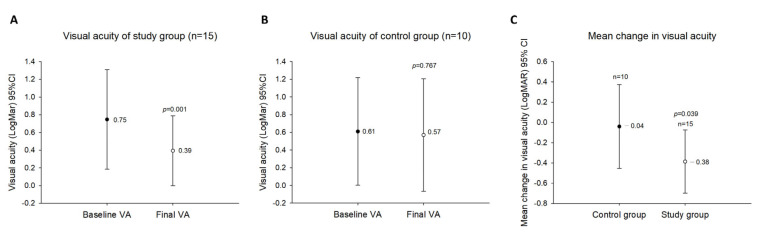
Changes in visual acuity during the follow-up period. (**A**). The visual acuity of the study group. (**B**). The visual acuity of the control group. (**C**). Comparison between the study group and the control group regarding changes in mean visual acuity during the follow-up period.

In the study group, visual acuity ameliorated significantly (*p* = 0.001) from 0.75 ± 0.56 logMAR preoperatively to a best postoperative visual acuity of 0.39 ± 0.39 logMAR (Figure 2A). Thirteen eyes (86.67%) revealed visual acuity amelioration, and two eyes (13.33%) were unchanged during the follow-up course in comparison with baseline measurements. After successive surveys, only three eyes (30%) revealed visual acuity amelioration in the control group. Six eyes (60%) had unchanged visual acuity during the follow-up compared with baseline measurements. For one eye (10%), visual acuity deteriorated compared with baseline measurements. Visual acuity was significantly improved postoperatively in the study group (*p* = 0.001). In contrast, in the control group, the baseline measurements of best-corrected visual acuity (0.61 ± 0.61 logMAR) and best-corrected visual acuity during the follow-up (0.57 ± 0.64 logMAR) did not differ significantly (*p =* 0.767) (Figure 2B). In addition, the differences in visual acuity changes between these two groups were statistically significant (F = 0.027, *p =* 0.039) (Figure 2C).

**Figure 3:**
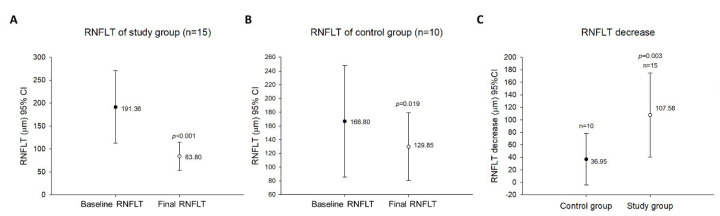
Changes in RNFL thickness during the follow-up period. (**A**). Optical coherence tomography measurement of the study group revealed clinical remission in disc edema postoperatively. (**B**). The RNFL thickness of the control group, measured by optical coherence tomography. (**C**). Comparison between the study group and the control group regarding changes in mean RNFL thickness during the follow-up period.

The postoperative disc edema in the study group, which was measured by OCT, indicated clinical amelioration (*p* < 0.001). The preinjection RNFLT ranged from 86.75 μm to 320 μm (mean, 191.36 ± 79.04 μm). The final RNFLT ranged from 57 μm to 171 μm (mean, 83.80 ± 30.72 μm), with a mean reduction of 56.21% in RNFLT (Figure 3A). Among the initial and final data in the control group, the RNFLT evaluated by OCT demonstrated significant resolution (*p =* 0.019). The baseline RNFLT ranged from 97 μm to 330 μm (mean, 166.80 ± 80.95 μm). The final RNFLT ranged from 66 μm to 196 μm (mean, 129.85 ± 49.29 μm), with an average decrease of 22.15% in the RNFLT (Figure 3B). Furthermore, the variation in RNFLT significantly differed between these groups (F = 5.507, *p =* 0.003) (Figure 3C).

**Figure 4:**
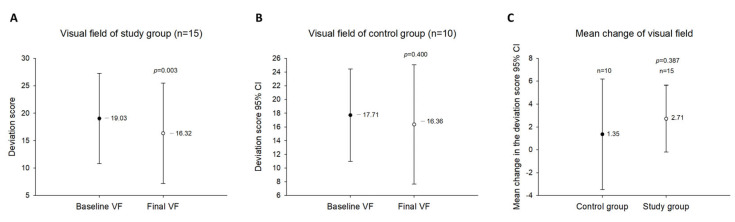
Changes in the visual field during the follow-up period. (**A**). The Humphrey visual field mean deviation score of the study group. (**B**). The Humphrey visual field mean deviation score of the control group. (**C**). Comparison between the study group and the control group regarding changes in the Humphrey visual field mean deviation score during the follow-up period.

The Humphrey visual field mean deviation score in the study group improved significantly (*p* = 0.003) from −19.03 ± 8.24 dB preoperatively to −16.32 ± 9.14 dB postoperatively (Figure 4A). When comparing the follow-up measurements and the baseline measurements, twelve eyes (80.00%) revealed visual field improvement, and three eyes (20.00%) deteriorated. In contrast, the Humphrey visual field mean deviation score in the control group did not differ significantly (*p =* 0.400) between baseline (−17.71 ± 6.73 dB) and follow-up (−16.36 ± 8.69 dB) (Figure 4B). Additionally, the differences between these two groups concerning changes in the visual field were not statistically significant (F = 0.724, *p =* 0.387) (Figure 4C).

### 3.3. Adverse Events

The study group reported no obvious complications, such as elevated intraocular pressure or cataract progression, postoperatively or during follow-up. Moreover, no severe complications occurred, such as postoperative endophthalmitis or retinal detachment.

## 4. Discussion

Nonarteritic anterior ischemic optic neuropathy (NAION) is the most prevalent form of optic nerve swelling and optic neuropathy in individuals aged 50 years and above [30]. Several risk factors have been strongly associated with NAION, including hypertension, hypercholesterolemia, diabetes mellitus, and cardio- and cerebrovascular diseases, as well as obstructive sleep apnea [31].

Although the exact pathogenesis of NAION remains uncertain, the prevailing hypothesis suggests that it is primarily triggered by hypoperfusion of the short posterior ciliary arteries that supply the optic nerve. This hypoperfusion leads to ischemia, resulting in swelling of the optic nerve segment that traverses through a narrow opening in the scleral canal [31]. Consequently, compartment syndrome develops, affecting adjacent axons that become compressed within the confined space of the scleral canal’s small opening. This compression ultimately leads to apoptosis and the demise of ganglion cells, which constitute the optic nerve’s axons.

Spontaneous visual acuity amelioration was observed in 42.7% of patients with NAION after six months of follow-up in the IONDT [14]. In contrast, there was still no visual amelioration in 44.9% of patients, and further visual exacerbation was found in 12.4% of patients who were followed up. Thus, it is worthwhile to look for a valid therapeutic approach to improve visual outcomes. In recent clinical applications, anti-VEGF drugs have been widely applied in neovascular lesions, such as neovascular glaucoma and macular edema associated with retinal vascular diseases, such as age-related macular degeneration and diabetic retinopathy.

VEGF is a potent angiogenic factor that affects vascular permeability. Intravitreal anti-VEGF medications have been used in patients with acute NAION by some physicians. It is believed that anti-VEGF agents may have two beneficial functions in the disease course of NAION. One function is the decrease in contingent subretinal fluid when arising, and the second is the decrease in optic disc edema by reducing capillary permeability [32]. The administration of anti-VEGF agents leads to a decrease in capillary permeability, resulting in accelerated resolution of optic disc edema. By reducing compression on capillaries within the optic nerve head and improving blood flow, the function of ischemic axons that are still viable but nonfunctional could potentially be restored. Because acute disk edema may lead to further axonal damage in NAION via a vicious cycle of ischemia resulting in edema and compartment syndrome, many medical and surgical therapeutic strategies aim to shorten the period of disc edema to prevent further axonal ischemia and decrease neuronal death [33,34]. However, there is no generally accepted therapy for NAION thus far.

Some studies have reported the efficacy of anti-VEGF therapies in patients with new-onset NAION, with opposite results. Bennet and colleagues [35] presented their results from one patient with acute NAION who received intravitreal bevacizumab. Rapid resolution of optic disc edema and improved visual outcome were observed nine days postoperatively. Pece and associates [36] described three patients treated with one intravitreal injection of ranibizumab who had a 1–2-day history of NAION. Early remission of optic disc swelling one week after intravitreal injection was found in all patients. However, visual acuity or visual field improvement did not occur together with anatomical amelioration. Saatci and colleagues performed a retrospective patient study of 17 eyes of sixteen patients with new-onset NAION within 15 days (range, 2–15 days) and investigated the efficacy of one intravitreal ranibizumab injection [37]. One year after the injection, visual improvement was found in 14 of 17 eyes, no change in visual acuity was found in one eye, and decreased visual acuity was found in two eyes. Visual fields were ameliorated in nine eyes and unchanged in eight eyes. The mean RNFLT enormously declined after the injection during the follow-up in all patients. Bajin et al. reported four patients with acute NAİON who received a single intravitreal ranibizumab injection and suffered a visual decline period of 15 days or fewer with a follow-up of three months [38]. Visual improvement was noted in all patients. The researchers noted a significant resolution in the average RNFLT in all eyes evaluated with spectral-domain optical coherence tomography. During the follow-up course, no complications related to the injections were observed.

In a nonrandomized controlled clinical trial of patients with NAION, Rootman et al. [39] investigated the effects of the intravitreal injection of 1.25 mg of bevacizumab among 25 patients (of which 17 eyes received treatment and eight were assigned to the control group). The data revealed no significant discrepancy between the treatment and control groups regarding changes in the visual field, visual acuity, or optic nerve OCT thickness. Furthermore, two patients in the treatment group experienced a second NAION event in the same eye during the follow-up period. Additionally, other intravitreal drugs have been reported to treat NAION. Only one case report about another anti-VEGF agent, aflibercept, reported promising visual outcomes in a patient with unilateral NAION [29]. The visual acuity increased from 1/10 to 7/10, the mean RNFLT decreased from 182.4 µm to 159.7 µm, and the visual fields markedly improved postoperatively.

According to PubMed searches and to our knowledge, our study is the first retrospective study to compare intravitreal aflibercept with natural disease history in the management of patients with NAION. Our findings demonstrated that intravitreal injection of aflibercept might change the course of acute NAION regarding the natural history of the disease. The main outcome measures, such as visual acuity, RNFLT (an index of optic disc edema), and Humphrey visual field, were significantly affected by intravitreal aflibercept injection. Our study showed that the changes in visual acuity and mean RNFLT differed significantly between the study and control groups. However, the differences between the two groups concerning changes in the visual field were not statistically significant.

During the follow-up course, the average RNFLT greatly declined after the injection. After intravitreal injection of anti-VEGF, the fast remission of disc swelling and rapid amelioration in visual acuity imply that VEGF-mediated vascular permeability may play a role in tissue injury in NAION.

However, there are some controversial opinions about using an intravitreal anti-VEGF agent for patients with acute NAION. First, anti-VEGF agents may cause acute NAION [40,41,42,43]. Mansour et al. postulated that possible theories might consist of the deterioration of systemic hypertension due to the stress of the intravitreal injection, an increase in intraocular pressure owing to the procedure, and the vasoconstrictor function of anti-VEGF drugs [43]. Second, it is still unknown which anti-VEGF medication is more valid for new-onset NAION. The third controversy concerns the appropriate time point for injection after the acute event. This implies that the therapeutic window for new-onset NAION may be sustained for two to three weeks based on animal experimental data [32].

The major limitations of our investigation are the small sample size in both the study and the control groups, the retrospective study design, the lack of randomization, and the short follow-up period. To evaluate the long-term therapeutic effect and safety of intravitreal aflibercept in patients with acute NAION, large prospective randomized clinical studies are warranted.

## 5. Conclusions

In conclusion, intravitreal injection of 2 mg of aflibercept seems to be beneficial for the treatment of NAION patients in the acute phase. Intravitreal injection of aflibercept can quickly relieve edema of the optic disc and lead to improved vision. Additionally, although the visual field test improved after intravitreal injection of aflibercept, there was no statistically significant difference between the control group and the study group (*p =* 0.387). A single injection is usually sufficient to reduce optic disc edema without complications. Based on the results of our study, we suggest that intravitreal injection of aflibercept could be an alternative treatment option for the treatment of acute NAION. Although our results seem satisfactory, the number of patients in the present study is too small to draw appropriate conclusions. Therefore, prospective trials with more patients, longer follow-up times, and appropriate control groups are needed to find the best plan for managing patients with acute NAION.

## Figures and Tables

**Table 1:** Baseline clinical characteristics.

	Study Group	Control Group	*p*-Value ^1^
Number	15 patients (15 eyes; 8 right eyes)	10 patients (10 eyes; 6 right eyes)	
Male:female	9:6	5:5	0.622
Mean age (years)	61.20 ± 12.36	60.40 ± 8.66	0.861
Mean follow-up period (days)	229.73 ± 43.44	258.90 ± 62.42	0.180
Baseline visual acuity (LogMAR ^2^)	0.75 ± 0.56	0.61 ± 0.61	0.570
Baseline RNFL thickness (μm)	191.36 ± 79.04	166.80 ± 80.95	0.462
Baseline mean deviation (dB)	−19.03 ± 8.24	−17.71 ± 6.73	0.677

^1^ The *p*-values were calculated using the independent-samples *t*-test (for age, mean follow-up period, baseline visual acuity, baseline RNFL thickness, and baseline mean deviation) and chi-square test (for sex). ^2^ LogMAR: logarithm of minimal angle of resolution.

## Data Availability

Not applicable.
